# Implant abutment using hand drivers versus torque wrench

**DOI:** 10.6026/97320630019221

**Published:** 2023-02-28

**Authors:** Himani Singh, Subhash Sonkesriya, Bhumika J Patel, Varsha Rathod, Bharat Gupta, Tarun Vyas, Anshul Sawhney, Ramanpal Singh Makkad

**Affiliations:** 1 Department of Oral Medicine and Radiology, Kothiwal Dental College and Research Centre, Moradabad, Uttar Pradesh, India; 2 Department of Prosthodontics, Government College of Dentistry, Indore, M.P., India; 3 Department of Oral Medicine and Radiology, Gujarat, India; 4 Department of Periodontology, Bharati Vidyapeeth Dental College and Hospital, Navi Mumbai, India; 5 Department of Periodontology, MGM Dental College, Navi Mumbai, India; 6 Department of Oral Medicine and Radiology, Vyas Dental College and Hospital, Jodhpur, Rajasthan, India; 7 Department of Dentistry, MSDASMC Bahraich, India; 8 Department of Oral Medicine and Radiology, New Horizon Dental college and Research institute, Bilaspur, Chhattisgarh, India

**Keywords:** Dental implants, abutment screw, hand drivers, internal thread, torque wrench

## Abstract

The most frequent instrument used to begin tightening screws is a manually regulated screwdriver. Regarding manually regulated screwdrivers, predicted margins of error vary between fifteen percent to forty eight percent. Mechanical Torque restricting devices can consistently produce the requisite torques. As a result, devices like wrenches are needed to achieve the desirable values of torque. Hence, the present study was designed to evaluate the torque difference between handheld drivers and torque wrench and thereby its effect on the internal threads of implant surface.120 blocks was prepared from an autopolymerizing type of acrylic material each with a dimension of 1 inch. The centre of each block was affixed with analogue of dental impalnts with dimensions of 3.5 mm width and 13 mm length. With 60 specimens each, these models were split into two categories: hand torque specimens category and torque wrench specimens category. A stereomicroscope was used to look at the implant analog's internal threading architecture at a magnification of 100. At the bottom and top, four threads were spaced apart by a certain amount. Biowizard software was used for the assessment, and the results were recorded. Threads on the internal surface of dental implants were produced once more following torquing the implant's impression, and the stereomicroscope was used to quantify the separation between the 4 threads. Statistics were used to correlate the readings. All study participants' hand torque as well as torque wrench measurements were documented and statistical analysis was performed on them. When there was statistical analysis of the measurements then it was observed that mean values of torque in specimens included category of manual torque application was found out to be 33.6 ± 6.510 Ncm. On the other hand the mean values of torque in specimens included in category of torque application by torque wrench were found out to be 33.57 ± 3.472 Ncm. The outcome showed operator heterogeneity for both categories and operator variance when using a manual driver to generate torque. One independent - sample t test was used to contrast the mean data between the two categories, and P< 0.05 was chosen to determine whether the intergroup difference was meaningful. Because the torque values obtained with hand tightening were uneven, it may be concluded that different levels of hand torquing skill caused the torque to fluctuate. The torque wrench device displayed the desired torque data in the range that the manufacturer had advised. However, utilising manual drivers and a mechanical torque instrument did not cause any modifications to thread on the internal surface, and it rarely underwent significant deformation during the preliminary tightening torque readings. Thus, given that manually hand regulated drivers create a range of torques, it may be inferred that the employment of mechanical torque restricting instruments should be required.

## Background:

One of the leading reasons for a dental implant based restoration getting unsuccessful is the loosening of screw of abutment. [1] In everyday practice, prosthetic parts are repeatedly torqued when taking impressions, adjusting temporary replacements, and constructing the actual prosthesis. The practitioner is in charge of ensuring that the screw is tightened with the proper tension and highlights the significance of using a standardized torque wrench. However, some dental professionals prefer to employ hand regulated drivers in place of torque wrenches, resulting in an undefined torque. Additionally, when the dental implant abutment is adjusted, the threading present on internal surface of dental implants and dental screw may become distorted. Abutment components and superstructure interconnections in dental implants should be strong, and the sustainability of every one of these parts should be checked at successive recalls, according to established dental implant prosthodontic protocols. [2] Inadequate fit and tightness could be the cause of issues with loosening of screw of abutment and subsequent fracture. [3] For an implant based prosthesis to last a long period of time, the interfacial surface between implants and abutment implant abutment must be adequately protected. According to Huynh-Ba et al. [4], the implant based prosthesis had a five year prosthesis longevity possibility close to 95.8%. Even though implants have a significant success rate, they are nonetheless susceptible to a variety of problems, with mechanical difficulties happening in sixty percent to seventy percent of instances. Loosening of screw in dental implants was the mechanical fault that was documented the most frequently, occurring at an expected annual frequency of 2.1 percent to 10.4 percent and 20.8 percent across 5 years follows up and 10 years follow up, respectively. Screw resting, the amount of the applied functional load, and the incapacity to apply enough tightened force torque to the dental screw are a few variables that might contribute to loosening of screw. Making sure that screws are tightened properly is one of the easiest methods for avoiding loosening of screw. [4, 5] According to model, the suggested sealing torque might vary between twenty to thirty Ncm, with 32 Ncm considered as the best torque. The most frequent instrument used to begin tightening screws is a manually regulated screwdriver. Regarding manually regulated screwdrivers, predicted margins of error vary between 15 to 40%. Mechanical Torque restricting devices (MTLDs) can consistently produce the requisite torques. [6]As a result, MTLDs like wrenches are needed to achieve the desirable values of torque. Hence, the present study was designed to evaluate the torque difference between handheld drivers and torque wrench and thereby its effect on the internal threads of implant surface. The null hypothesis is that no difference could be found using a handheld drivers and a torque wrench.

## Materials and methods:

120 blocks were prepared from an autopolymerizing type of acrylic material each with a dimension of 1 inch. The centre of each block was affixed with analogue of dental implants with dimensions of 3.5 mm width and 13 mm length. The implants used in study were marketed by ADIN Limited, Israel. With 60 specimens each, these models were split into two categories: hand torque specimens category and torque wrench specimen's category. Clamps were used to fasten these specimens to the table. Manual torque specimens were clamped at right side whereas torque wrench specimens were clamped at left side. The study had 60 doctors in total - 40 men and 20 women. All participants' ages varied from 23 years to 37 years, with a mean age of twenty years.

##  Comparison of finger administered driver and wrench torque variations in threading pattern on internal surface of implant:

The interior area of the implant analogue was dried by airflow only after study templates were fixed. Light body component of elastomeric impression matter was put into the intra - oral end of an impression capsule, which was then introduced as thoroughly as feasible within the body of dental implant. The light body impression substance was inserted up till it got overflowed from shoulder of dental implant. The impression substance had an interproximal hardwood wedge placed in the centre and subjected to polymerize. Swinging the hardwood wedge in a anticlockwise manner gently removed the impression matter from the inside face of dental implant. Next, a stereomicroscope was used to look at the implant analog's internal threading architecture at a magnification of 100. At the bottom and top, four threads were spaced apart by a certain amount. Biowizard software was used for the assessment, and the results were recorded. Threads on the internal surface of dental implants were produced once more following torquing the implant's impression, and the stereomicroscope was used to quantify the separation between the 4 threads. Statistics were used to correlate the readings.

## Comparison of finger controlled driver and mechanical device like torque wrench:

With a precision of 0.1 Ncm, an electronic torque gauge was applied to calculate torque between 10 Ncm and 200 Ncm. The gauge can be physically regulated. It is 203 mm long and has a 0.25 inch bit terminal connection with a self-lock bit retainer. This 1.27-inch hex tip of the implant system's 1/4-inch bit end was modified with an adaptor to accommodate the handheld driver as part of the torque measurement system that also uses spring-type torque wrenches.2 consecutive abutments (ADIN, Israel) were required to be torqued by each registrant, one utilising a manually tightened hex torque implant driver and the other employing a torque wrench. Screws of dental implant were adjusted employing the similar driver 10 minutes following the first torque delivery to lessen the settling impact. The hex driver then was connected with the converter to the electronic screwdriver to quantify the maximum fastening output, which was given in Ncm, after the persons had manually torqued the dental implant abutment. The torque measurements underwent evaluation and documentation. The study participants were shown how to torque the dental implant abutment employing a torque wrench in a comparable pattern. After applying torque, the wrench was withdrawn, and the hex was again connected to the electronic screwdriver with an adaptor to measure the highest torque value. As a result, all study participants' hand torque as well as torque wrench measurements were documented and statistical analysis was performed on them.

## Statistical Analysis:

Utilizing the SPSS version 20.0 and a significance threshold of P = 0.05, the torque measurements of 60 users using manually regulated drivers and employing torque wrench had been documented and analytical analysis was conducted. Student t test and one way ANOVA was used for statistical analysis.

## Results:

When there was statistical analysis of the measurements then it was observed that mean values of torque in specimens included category of manual torque application was found out to be 33.6 ± 6.510 Ncm. On the other hand the mean value of torque in specimens included in category of torque application by torque wrench was found out to be 33.57 ± 3.472 Ncm. It was noticed that values of SD was 6.510 in specimens included in category of manual torque application which was three times as compared to SD values of 2.472 in specimens included category of torque application by torque wrench. The range of values of torque was 29-36 in specimens included in category of torque application by torque wrench. With maximum value of 36 and minimum value of 29 while range was 28-44 in specimens included category of manual torque application with maximum value of 44 and minimum value of 28. The outcome showed operator heterogeneity for both categories and operator variance when using a manual driver to generate torque. One independent - sample t test was used to contrast the mean data between the two categories, and P< 0.05 was chosen to determine whether the intergroup difference was meaningful.

The mean post-treatment torque values in specimens included in category of manual torque application was 872.144 ±plusmn;5.117 and mean post-treatment torque values in specimens included in category of torque application by torque wrench was 875.461±4.213.The difference in post treatment torque values in both categories was 3mm. The pretreatment torque values for both categories were 861. The difference in pretreatment and post treatment torque values was approximately 11 in category of manual torque application and 14 in category of torque application by torque wrench. The findings when analysed statistically using one way ANOVA was significant statistically. (p<0.01). (Table 2)

## Discussion:

When obtaining impressions, correcting temporary replacements, and building the actual prosthesis, prosthetic parts are often torqued. The practitioner emphasizes the need of using a standardized torque wrench and is in responsibility of making sure the screw is tightened with the appropriate tension. However, some dentists would rather use hand-regulated drivers as opposed to torque wrenches, which lead to an ill-defined torque. The internal threading of dental implants and dental screws may also get deformed when the dental implant abutment is modified. [7,8] Finger operated screwdrivers are the most widely used instrument for beginning to tighten screws. The expected margins of error for screwdrivers that are manually controlled are higher. The necessary torques can be reliably generated using torque restriction devices [9]. To attain the desired torque values, tools like wrenches are also required. Therefore, the goal of the current investigation was to determine how the internal threads of the implant surface would be affected by the torque differential between fingers controlled drivers and torque wrenches. The underlying assumption was that there would be no difference between a mechanically engineered driver and one that could be controlled with a finger [10, 11]. The findings of our investigation indicated that manual tightening can result in values of torque that are higher than those recommended by the manufacturer. The information demonstrated that the maximal mean torque produced by manually used drivers exceeded that produced by a torque wrench. Since a substantial change was discovered when using a manually regulated torque driver as compared to torque wrench therefore the null hypothesis was discarded. In this study when there was statistical analysis of the measurements then it was observed that mean values of torque in specimens included category of manual torque application was found out to be 33.6 ± 6.510 Ncm. On the other hand the mean values of torque in specimens included in category of torque application by torque wrench were found out to be 33.57 ± 3.472 Ncm. It was noticed that values of SD was 6.510 in specimens included in category of manual torque application which was three times as compared to SD values of 2.472 in specimens included category of torque application by torque wrench. The range of values of torque was 29-36 in specimens included in category of torque application by torque wrench. With maximum value of 36 and minimum value of 29 while range was 28-44 in specimens included category of manual torque application with maximum value of 44 and minimum value of 28. The outcome showed operator heterogeneity for both categories and operator variance when using a manual driver to generate torque. One independent - sample t test was used to contrast the mean data between the two categories, and P< 0.05 was chosen to determine whether the intergroup difference was meaningful. According to Gross et al. [9] mechanical torque generators are essential to provide the necessary torque since hand regulated torque drivers exhibit high variability among different individuals. In a research by Gross et al. [9] the mean values of torque obtained in both types of torque applying instruments was comparable to our study. Although implants have a high success rate, they are nonetheless prone to a number of issues, with mechanical issues occurring between 60% and 70% of the time. The mechanical issue that was recorded the most commonly was loosening of screw in dental implants, which occurred at an estimated annual frequency of 2.1 percent to 10.4 percent and 20.8 percent during 5 years follow up and 10 years follow up, respectively. A few factors that could lead to dental screw loosening include screw resting, the amount of functional load applied, and the inability to impart sufficiently tightened force torque to the dental screw. One of the simplest ways to prevent screw loosening is to make sure that screws are correctly tightened. [5] The recommended sealing torque may range from twenty to thirty Ncm depending on the model, with 32 Ncm being the ideal torque. Siamos et al. [6] as well as Hill et al. [10] both found significant individual variation in the torque delivered by screwdrivers. Comparable findings were confirmed in our investigation, which also showed that certain people could produce higher torque than the maximum intended target number of the majority of manufacturers of dental implants i.e. 32 Ncm. Clinical professionals should therefore be worried about both reduced as well as elevated tightening of components of implants.[12, 13, 14, 15] Dental implant manufacturers must be more specific about the suggested torque value and the maximum permitted torque numbers for all elements due to the variation in torquing skills across dentists. As determined from our study, telling clinicians to "fingertip tightens alone" is insufficient because this could result in variations of between 27 and 43 Ncm. According to recognised dental implant prosthodontic procedures, abutment components and superstructure interconnections in dental implants should be robust, and the durability of each of these pieces should be examined at subsequent recalls. [16, 17] Problems with the abutment screw loosening and resulting fracture may be due to inadequate fit and tightness. [18, 19] The interfacial surface between implants and abutment implant abutment must be appropriately preserved for an implant-based prosthesis to last for a long time. The implant-based prosthesis had a five-year prosthesis longevity potential close to 95.8%, according to Huynh-Ba et al. [4]. In this study the interior of the implant analogue was dried by airflow in this investigation, but only after the study templates had been fixed. The intra-oral end of an impression capsule received a light body component of elastomeric impression material, which was subsequently inserted as thoroughly as possible within the body of the dental implant. The thin body impression material was put all the way to the dental implant's shoulder before spilling over. An interproximal hardwood wedge was positioned in the centre of the impression material and allowed to polymerize. The impression material from the inside face of the dental implant was gently removed by gently rotating the hardwood wedge anticlockwise. The internal threading architecture of the implant analogue was then observed under a stereomicroscope at a 100-fold magnification. Four threads were spaced a specific distance apart at the bottom and top. The evaluation was conducted using the Biowizard programme, and the outcomes were documented. After torqueing the implant's impression, threads on the internal surface were created once more, and the stereomicroscope was utilised to measure how far apart the four threads were from one another. The readings were correlated using statistics. When there was analysis of variations in the architecture of threading on the inner aspect of implant and screw then the mean of pretreatment torque values was 850 mm. The posttreatment hand torque was recorded to be 861.033 ± 4.006 mm whereas the post treatment torque wrench value was 864.350 ± 3.102 mm, with the mean difference between the two groups being 14.35 ± 3.103 mm. A difference of 11 mm and 14 mm was noticed for posttreatment hand torque and wrench values, respectively, compared to pretreatment torque values. However, there was only 3 mm difference between the posttreatment hand torque and posttreatment torque wrench values. The mean values were compared and subjected to statistical analysis using ANOVA statistical analysis. The level of significance was found to be statistically significant (P < 0.05). The deformation of abutment screws has been the subject of substantial study, but little is known about alterations to threads on internal surface of dental implants. [20] When the abutment screw is tightened and loosened repeatedly, Novman et al. [19] examined the surface alterations of the threads on the internal surface of dental implants and screws and came to the conclusion that there had been no alteration to the threads present on the internal surface of implants.. According to a research by Guzaitis et al. the surface modifications affecting implants were less than those seen on the screw of prosthesis because implant alloy toughness is higher than toughness of screw of prosthesis. [20, 21, 22, 23]

## Conclusions:

Because the torque values obtained with hand tightening were uneven, it may be concluded that different levels of hand torquing skill caused the torque to fluctuate. The torque wrench device displayed the desired torque data in the range that the manufacturer had advised. However, utilising manual drivers and a mechanical torque instrument did not cause any modifications to thread on the internal surface, and it rarely underwent significant deformation during the preliminary tightening torque readings. Thus, given that manually hand regulated drivers create a range of torques, it may be inferred that the employment of mechanical torque restricting instruments should be required.

## Figures and Tables

**Table 1:** Comparison of hand driver and torque wrench values using one sample t-test test

**Categories**	**Mean±SD**	**t-test**	**Degreeoffreedom**	**P**	**Range**
Handtorquing	33.728±6.510	34.139	31	0.005*	28-44
Torquewrench	33.574±2.472	76.423	31	0.004*	29-36

**Table 2:** Comparison of internal threads changes of hand and wrench torque values using ANOVA statistical test

**Torque**	**Mean±SD**	**ANOVA**	**Degreeoffreedom**	**p**
Posthandtorque	872.144±5.117			
Postwrenchtorque	875.461±4.213	13.964	61	0.002*
Pretorque	861			
